# 

**Published:** 1881-11

**Authors:** 


### EXAMINERS' MEETING.

The Annual Meeting of the Board of Examiners, acting under the law regulating the practice of Dentistry in the State of Ohio, will meet in the City of Columbus on Wednesday, Dec. 7, at 12 o'clock, M., 1881, in the American House. All interested should give attention to this notice, and be promptly present at the time designated.

### OHIO STATE DENTAL SOCIETY.

The Annual Meeting of the Ohio State Dental Society will be held in Columbus, on Wednesday, Dec. 7th, and continue till Friday P. M. or evening. It is important that there should be a large attendance.

The financial embarrassments under which the Society labored for several years have all been removed by the payment of all its liabilities. The treasury is in a healthy condition.

The Society is now ready for a career of work and usefulness greater than ever before. It is desirable, that there be a large accession to the membership. There is a large number of most excellent men in the State who are not members of this body who owe it to the public, to the profession and to themselves, to unite with and work in it. There should be an attendance of from two to three hundred. Let every one who possibly can, feel urged to prepare something in the way of papers, cases, or appliances, that may add interest and be profitable.

There is much of good, in various ways, to be attained in such meetings. Often men have said "That the attainment of a single item of knowledge was far more than compensation for the expense and time incurred in attending the meetings." And this can be fully accomplished for any one who may attend, if all will do their duty.

Then, again, the social advantages are by no means of minor importance. As we become acquainted, and know each other well, we perceive and appreciate qualities that otherwise would remain hidden, and that which we may have imagined as objectionable becomes less, or fades away altogether. Those engaged in a similar pursuit or calling will, under favorable circumstances, have a common feeling and sympathy, which, by proper use, will tend to unite the profession in an harmonious whole.

Now let any dentist in the State who appreciates and values his chosen profession, resolve himself into a committee of one, whose duty it shall be, in the first place, to secure his own attendance at the meeting, and, secondly, the attendance of his professional neighbors.

Now, brothers, if you will accept and act upon these suggestions, we will have in Dec. next the largest and best meeting of the Ohio State Dental Society ever held. So mote it be.

### REMOVAL.

The Editorial office of the Dental Register, and the Professional office of J. Taft, and Wm. Taft, has been removed from No. 117 West Fourth Street to No. 122 West Seventh Street, where it will be permanently located.

### THE OHIO STATE DENTAL SOCIETY.

The above Society will hold its Sixteenth Annual Session at Columbus, Wednesday, Thursday and Friday, Dec. 7, 8 and 9, 1881. The profession from other States are invited to participate in the meetings. A full attendance of members is desired, as business of importance will come before the Society.

### SUBJECTS FOR DISCUSSION.

I. Preparation of cavities for gold fillings.
II. What shall be done with the first permanent molars?
III. Methods of mounting artificial crowns on roots of teeth.
IV. Diseases—causes and treatment—of the maxillary antrum.
V. Histological structure of the teeth.
VI. Oral surgery.

W. H. SILLITO, Xenia, O.,
Recording Secretary.

### CONTENTS.

### COMMUNICATIONS.

### THE DENTAL REGISTER,

A MONTHLY MAGAZINE DEVOTED TO THE INTERESTS OF

**THE DENTAL PROFESSION.**

*TERMS REDUCED TO $2.50 PER YEAR. SINGLE COPIES 25C.*

EDITED BY PROF. J. TAFT, D.D.S.

AND

***Published by SPENCER, CROCKER & CO., Ohio Dental Depot.***

The volume commences in January and closes in December.

The REGISTER being issued monthly is always fresh, therefore Indispensable to the practitioner who desires to keep posted up with the new inventions and improvements which are daily developed. Neither expense nor effort will be spared to give value and variety to its contents. Articles will be illustrated with well executed engravings whenever they will add to the interest or assist to explanation.

Its extensive circulation and frequency of issue make it one of the BEST DENTAL ADVERTISING MEDIUMS in the United States.

All subscriptions must begin with January or July.

Local or special notices, five lines or less, will be inserted for $3 each insertion; over five lines, 50 cts. per line.

All advertising bills payable in advance, or at furthest, after the issue of the first number containing the advertisement. All transient advertisements to be paid for in advance.

☞CONTRIBUTIONS SOLICITED.

Exclusively Editorial Communications should be addressed to Editor.

The latest number of the REGISTER may be ordered with or without other goods at any time, and all letters should be addressed to

***SPENCER, CROCKER & CO., Ohio Dental Depot, Cincinnati, O.***

